# Transcription Factor PLAGL1 Is Associated with Angiogenic Gene Expression in the Placenta

**DOI:** 10.3390/ijms21218317

**Published:** 2020-11-06

**Authors:** Rebekah R. Starks, Rabab Abu Alhasan, Haninder Kaur, Kathleen A. Pennington, Laura C. Schulz, Geetu Tuteja

**Affiliations:** 1 Genetics, Development, and Cell Biology, Iowa State University, Ames, IA 50011, USA; rstarks1@iastate.edu (R.R.S.); rabab@iastate.edu (R.A.A.); hkaur@iastate.edu (H.K.); 2 Bioinformatics and Computational Biology, Iowa State University, Ames, IA 50011, USA; 3 Obstetrics and Gynecology, Baylor College of Medicine, Houston, TX 77030, USA; Kathleen.Pennington@bcm.edu; 4 Obstetrics, Gynecology and Women’s Health, University of Missouri, Columba, MO 65212, USA; schulzl@health.missouri.edu

**Keywords:** RNA-seq, ChIP-seq, enhancers, transcription factors, placenta, PLAGL1, gestational diabetes, tube formation, blood vessel development

## Abstract

During pregnancy, the placenta is important for transporting nutrients and waste between the maternal and fetal blood supply, secreting hormones, and serving as a protective barrier. To better understand placental development, we must understand how placental gene expression is regulated. We used RNA-seq data and ChIP-seq data for the enhancer associated mark, H3k27ac, to study gene regulation in the mouse placenta at embryonic day (e) 9.5, when the placenta is developing a complex network of blood vessels. We identified several upregulated transcription factors with enriched binding sites in e9.5-specific enhancers. The most enriched transcription factor, PLAGL1 had a predicted motif in 233 regions that were significantly associated with vasculature development and response to insulin stimulus genes. We then performed several experiments using mouse placenta and a human trophoblast cell line to understand the role of PLAGL1 in placental development. In the mouse placenta, *Plagl1* is expressed in endothelial cells of the labyrinth layer and is differentially expressed in placentas from mice with gestational diabetes compared to placentas from control mice in a sex-specific manner. In human trophoblast cells, siRNA knockdown significantly decreased expression of genes associated with placental vasculature development terms. In a tube assay, decreased *PLAGL1* expression led to reduced cord formation. These results suggest that *Plagl1* regulates overlapping gene networks in placental trophoblast and endothelial cells, and may play a critical role in placental development in normal and complicated pregnancies.

## 1. Introduction

The placenta is a specialized organ playing a crucial role in the health of the fetus and mother during pregnancy, regulating the exchange of nutrients, gases, hormones and waste [1]. Inefficient transfer of materials from the mother to the fetus is frequently associated with adverse health outcomes for the fetus [2]. The placenta also plays a protective role, regulating the immune response of cells and preventing toxic elements from reaching the fetus [3,4]. Placental insufficiency, as well as many other placental abnormalities, have been attributed to misregulated gene expression [5,6]. Despite its importance, many aspects of placental development and the genetic mechanisms involved in its function remain unknown.

To elucidate these mechanisms, many researchers utilize the mouse model, which has contributed greatly to our understanding of the development of the placenta. Similar to humans, rodents have a single disc-shaped, hemochorial placenta [7] to sustain the fetus during development. In the human placenta, trophoblast cells invade deeply into the myometrium, and partially replace the endothelial wall of maternal spiral arteries, thereby channeling maternal blood into the intervillous spaces. Trophoblast lining the villi can then exchange factors between the maternal blood and fetal vessels running through the villous core [1]. Similarly, in the mouse, trophoblast cells replace the endothelial wall of maternal spiral arteries in the near decidua, channeling maternal blood through trophoblast-lined canals to spaces in the labyrinth, which are surrounded by trophoblast that exchange material between the maternal blood and the adjacent fetal vessels [8]. Labyrinth formation begins around embryonic day (e) 8.5 with the fusing of the chorion and allantois. Subsequently, the chorion begins to fold and form branches that become the trophoblast-lined maternal blood spaces, while allantoic offshoots form the interdigitating fetal vessels [9]. Thus, coordinated interactions between trophoblast and endothelial cells, with trophoblasts assuming key endothelial properties, are critical for placental development and function.

Regulation of these complex interactions requires transcriptional machinery to interact with the appropriate regulatory elements and cofactors in a spatiotemporal manner. A common cause of genetic misregulation is disruption of cis-regulatory elements, specifically in enhancers [10,11]. Despite the crucial role enhancers play in gene regulation, there remains a large deficit in our understanding of the gene-enhancer regulatory networks that are important for placental function [12], including trophoblast-endothelial cell interactions.

A previous study compared the enhancer landscape before (e7.5) and after (e9.5) chorioallantoic fusion in the mouse placenta, identifying an e7.5-specific cell migration network of transcription factors (TFs) and enhancers [13]. The goal of the present study is to identify e9.5-specific TFs and enhancers critical to placental development. In order to identify novel TFs that could be important for regulating processes in the mouse placenta after chorioallantoic fusion, we utilized publicly available H3k27ac ChIP-seq data, defining putative active enhancers in the e7.5 and e9.5 placenta, as well as RNA-seq data generated at the same timepoints [13]. We identified several highly expressed TFs that were upregulated in the e9.5 placenta. One of these TFs, pleiomorphic adenoma gene-like 1 (PLAGL1), has a motif highly enriched in e9.5-specific enhancers compared to e7.5-specific enhancers. Overexpression of *PLAGL1* has been associated with transient neonatal diabetes mellitus [14], while reduced expression has been found in female intrauterine growth restricted placentas, suggesting it plays an important role [15].

To further understand the functions of PLAGL1, we carried out additional experiments in mouse placenta and a human trophoblast cell line, HTR-8/SVneo, due to the high expression of *Plagl1* in both systems. By combining computational predictions with experimental validations, we were able to identify novel functions for PLAGL1 in two models frequently used to study human placenta, which could contribute to insights into placental development and certain pregnancy disorders.

## 2. Results

### 2.1. E9.5-Specific Genes and Enhancers Are Associated with Vasculature Development

**Figure 1:**
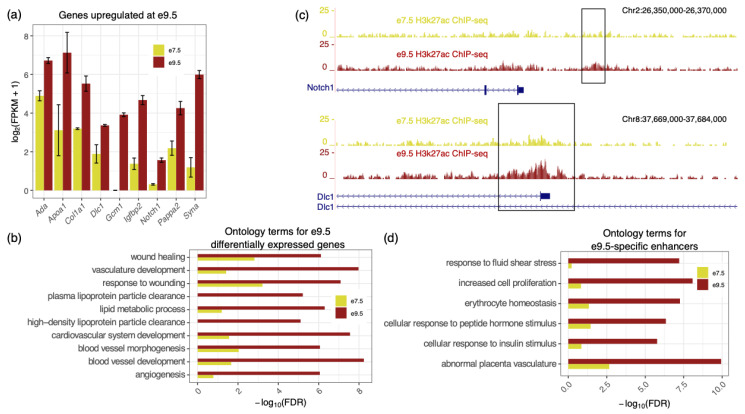
Genes and enhancers enriched in the placenta at e9.5 are associated with vasculature development. (**a**) Examples of genes significantly upregulated at e9.5 (FPKM ≥ 10, fold ≥ 2, and FDR ≤ 0.05) that are also associated with placental functions. (**b**) Top 10 GO biological process terms, according to GREAT, using the hypergeometric fold (≥2), FDR (≤0.05), and gene association (≥5 associated genes) cutoffs. Genes significantly upregulated at e9.5 are related to lipid metabolism, and vasculature development. (**c**) Examples of e9.5-specific enhancers and their corresponding H3k27ac activity at e9.5 and e7.5, shown using the UCSC genome browser. Boxes correspond to regions identified as e9.5-specific. (**d**) E9.5-specific enhancers are associated with genes involved in proliferation, response to hormones, and placenta vasculature development.

RNA-seq and H3k27ac ChIP-seq were previously carried out on mouse placenta at e7.5 and e9.5 to identify genes upregulated and enhancers specific to the e7.5 timepoint [13]. However, the genes upregulated at e9.5, as well as the enhancers more active at this timepoint were not thoroughly investigated. We first analyzed the 583 genes that were highly expressed and upregulated at e9.5 (fragments per kilobase of transcript per million of mapped reads (FPKM) ≥ 10, fold ≥ 2, and FDR ≤ 0.05). As expected, we see higher e9.5 expression of genes related to processes important in the e9.5 placenta including, *Apoa1* (lipid metabolism [16]); *Notch1*, *Col1a1*, and *Dlc1* (blood vessel development [17,18,19]); *Gcm1* (hormone production [20]); *Igfbp2*, *Pappa2*, *Ada* (fetal growth and development [21,22,23]); and *Syna* (trophoblast differentiation [24]) (Figure 1a). We then used the Genomic Regions Enrichment of Annotations Tool (GREAT) [25] to identify the biological processes associated with the highly expressed and upregulated e9.5 genes using the single nearest gene option. In general, we found that terms related to metabolism and vasculature development are more enriched in genes upregulated at e9.5 than genes upregulated at e7.5 (Figure 1b).

Next, we analyzed e9.5-specific enhancers. We observed that e9.5-specific enhancers were located near genes with known roles in the midgestation placenta (Figure 1c). For example, *Notch1* plays a role in promoting trophoblast differentiation, and regulates angiogenesis and placental branching [17,26]. *Dlc1*, a tumor suppressing gene, also contributes to the development of placental vasculature [27]. Ontology analysis of the e9.5-specific enhancers showed that many terms, such as ‘response to insulin stimulus’ and ‘abnormal placental vasculature’, are more significantly enriched in e9.5-specific enhancers compared to e7.5-specific enhancers (Figure 1d; Supplemental Table S1).

Since both upregulated genes and enhancers specific to e9.5 were associated with placental development terms, such as vasculature and labyrinth morphology, we next investigated which TFs could be regulating these processes.

### 2.2. Plagl1 Is Highly Expressed in the e9.5 Placenta and the PLAGL1 Binding Motif Is Enriched in e9.5-Specific Enhancer Regions

**Figure 2:**
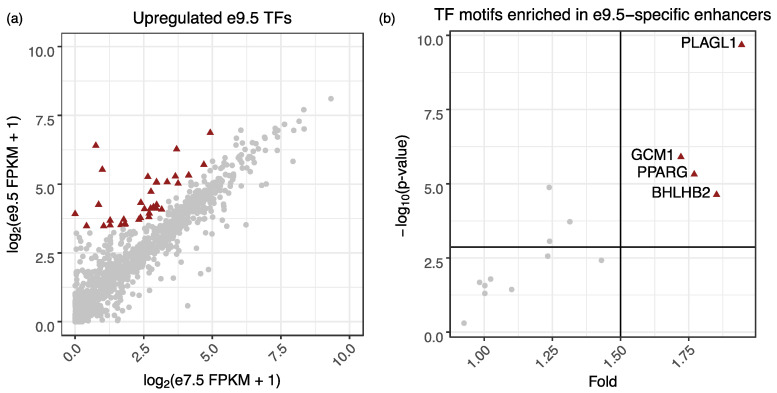
The PLAGL1 motif is enriched in e9.5-specific enhancers and is associated with blood vessel development genes. (**a**) Scatterplot showing the expression of all TFs at e7.5 and e9.5. Upregulated TFs at e9.5 are indicated by maroon triangles (fold ≥ 2, and *p*-value ≤ 0.05). (**b**) Of the 37 transcription factors upregulated at e9.5, four TFs (maroon triangles) pass the fold and Bonferroni corrected *p*-value thresholds (black lines).

To identify TFs that could be regulating e9.5-specific enhancers, we first determined which ones were upregulated at e9.5. Based on expression thresholds (FPKM ≥ 10), fold (≥2), and q-value (≤0.05), we identified 37 TFs upregulated at e9.5 (Figure 2a; Supplemental Figure S1a; Supplemental Table S2). We then used a phylofootprinting approach [28] to determine which of these TFs had motifs enriched in e9.5-specific enhancers. We ensured that the binding site predictions were conserved between the human and mouse genome since conserved binding sites are more likely to be functionally important [29]. Four TFs passed our motif fold (≥1.5) and *p*-value (≤0.05; Bonferonni correction) cutoffs: PLAGL1, GCM1, PPARγ, and BHLHB2 (Figure 2b). Interestingly, each of these TFs has a known role in the placenta, though some TFs are better studied. GCM1 is a well-known, important transcription factor expressed within labyrinthine trophoblast [30] that contributes to syncytiotrophoblast differentiation, chorionic branching [31], and hormone production [20]. PPARγ is also well-studied, known to play a variety of roles in the placenta including fatty acid uptake, differentiation, and vascularization [32,33,34]. BHLHB2 is expressed in cytotrophoblast and has been found to be upregulated in preeclampsia [35].

PLAGL1 had a high expression fold change (fold change: 46.93; e7.5 FPKM: 0.99; e9.5 FPKM 45.26) as well as the highest, most significant fold change among binding sites of the four TFs (Figure 2b). To confirm expression differences, we performed qPCR using e7.5 and e9.5 mouse placenta and found *Plagl1* was indeed significantly more highly expressed at e9.5 (*p*-value = 0.015) (Supplemental Figure S1b). PLAGL1 is an imprinted zinc-finger transcription factor known to play roles in regulating glucose uptake [36], apoptosis [37], and proliferation [38] and peaks in expression in the midgestation human placenta [39]. Although several functions of PLAGL1 have been identified, its role in placenta has not been thoroughly studied. Therefore, we performed additional experiments in both the mouse placenta as well as human trophoblast cells.

### 2.3. Plagl1 Is Expressed in Endothelial Cells of the Labyrinthine Layer

**Figure 3:**
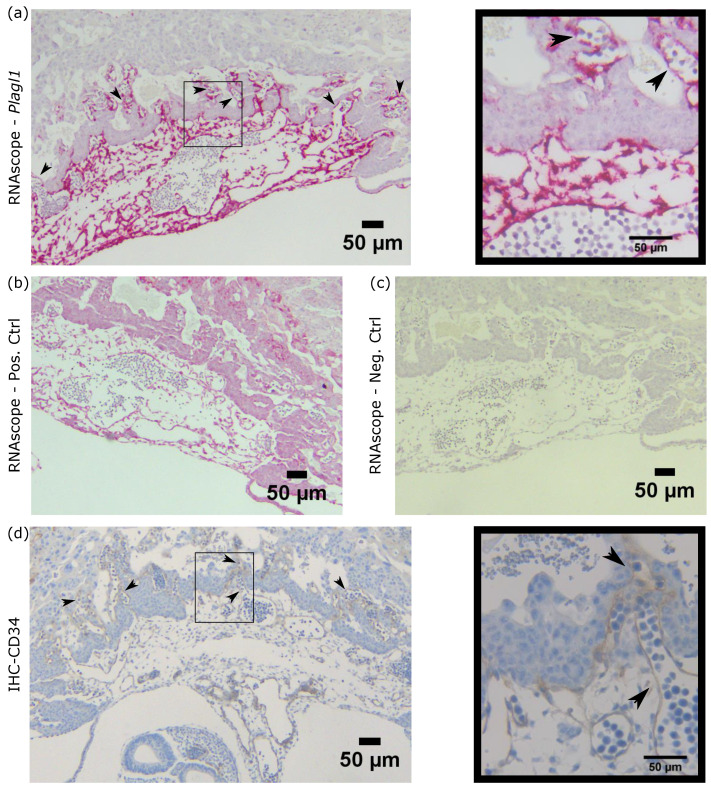
*Plagl1* is expressed within the developing labyrinth layer and allantois of the e9.5 mouse placenta. (**a**) RNAscope shows *Plagl1* RNA in the developing labyrinth as well as throughout the allantois (dark red) in the e9.5 placenta. Box shows zoomed in region (right). Arrowheads indicate *Plagl1* staining of endothelial cells forming fetal blood vessels. (**b**) RNAscope positive control staining *PPIB* within the placenta to determine RNA viability. (**c**) RNAscope negative control staining *DapB* within the placenta to determine nonspecific staining. (**d**) Immunohistochemistry shows CD34 staining of the vascular endothelial cells, displaying a similar pattern to *Plagl1* within the developing labyrinth. Box shows zoomed in region (right). Arrowheads indicate CD34 staining.

To determine where within the mouse placenta *Plagl1* is expressed we performed RNAscope with an e9.5 implantation site. *Plagl1* expression was observed within the developing labyrinth; specifically in the endothelial cells forming the fetal blood vessels (Figure 3a, Supplemental Figure S2a). Positive (*PPIB*) and negative (*DapB*) control probes were used for RNAscope to ensure *Plagl1* signal was not due to noise or unspecific binding (Figure 3b–c, Supplemental Figure S2b–c). Immunohistochemistry for CD34, a marker for vascular endothelial cells [40,41], was also performed and showed a similar staining within the labyrinth (Figure 3d; Supplemental Figure S2d). *Plagl1* is also expressed throughout the allantois, where fetal vasculature begins developing, eventually forming the endothelia in the labyrinth layer of the placenta [42], suggesting a role for PLAGL1 in vasculogenesis and labyrinth layer morphology.

### 2.4. Plagl1 Is Associated with Blood Vessel Development and Insulin Response

**Figure 4:**
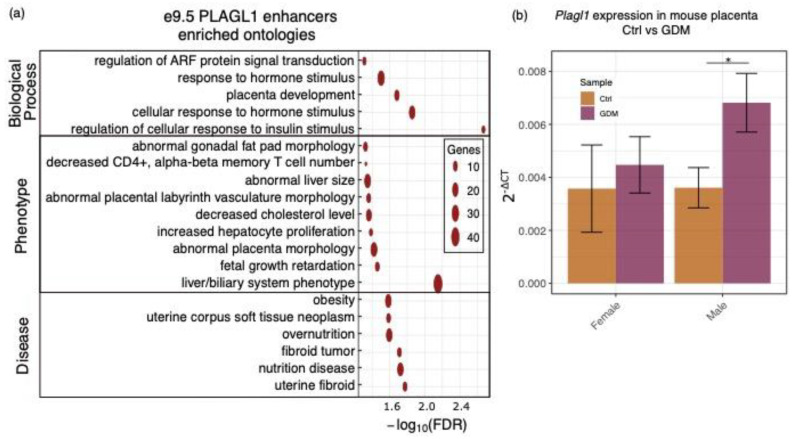
PLAGL1 is associated with placental morphology and *Plagl1* shows sex-specific differences in GDM mouse placentas. (**a**) The 233 e9.5-specific enhancers containing a PLAGL1 motif are associated with fetal growth, vasculature development, and other processes important in the placenta. (**b**) *Plagl1* is significantly overexpressed in the male placenta from mothers modeling GDM, but not in female placentas (*p*-value ≤ 0.05 (*)).

The PLAGL1 motif was identified in 233 e9.5-specific enhancers that were predicted to associate with genes involved in fetal growth, placental labyrinth morphology, and insulin response (Figure 4a). These enhancers were also predicted to associate with genes involved in obesity and overnutrition (Figure 4a). Interestingly, maternal gestational diabetes mellitus (GDM) has been associated with defective insulin signaling within the placenta [43] and increased vascularization [44], leading us to hypothesize that *Plagl1* could be misexpressed in GDM placentas.

To determine if *Plagl1* expression differs between control placentas and those from GDM mothers, we measured its gene expression in placentas from a mouse model for GDM [45]. Mice were fed a high-fat, high-sucrose diet a week prior to mating and throughout pregnancy resulting in glucose intolerance during pregnancy [45]. When comparing placentas from control mice and placentas from GDM mice at e17.5, we observed no significant difference in *Plagl1* expression (*p*-value = 0.07). Since the placental environment has been shown to affect males and females differently [46,47], and *PLAGL1* has also been shown to have sex-specific differences in fetuses [15], we analyzed the sexes separately. First, we compared *Plagl1* gene expression in control female placentas and control male placentas and found no difference (*p*-value = 0.98). Then, we compared *Plagl1* expression in GDM placentas to control placentas for each sex, and found significant upregulation of *Plagl1* in the GDM placentas in males only (*p*-value = 0.031) (Figure 4b; Supplemental Table S3). These findings indicate that GDM affects *Plagl1* in the placenta in a sex-specific manner in a murine model of GDM.

### 2.5. PLAGL1 Knockdown in the HTR-8/SVneo Cell Line Predicts a Role in Blood Vessel Remodeling

**Figure 5:**
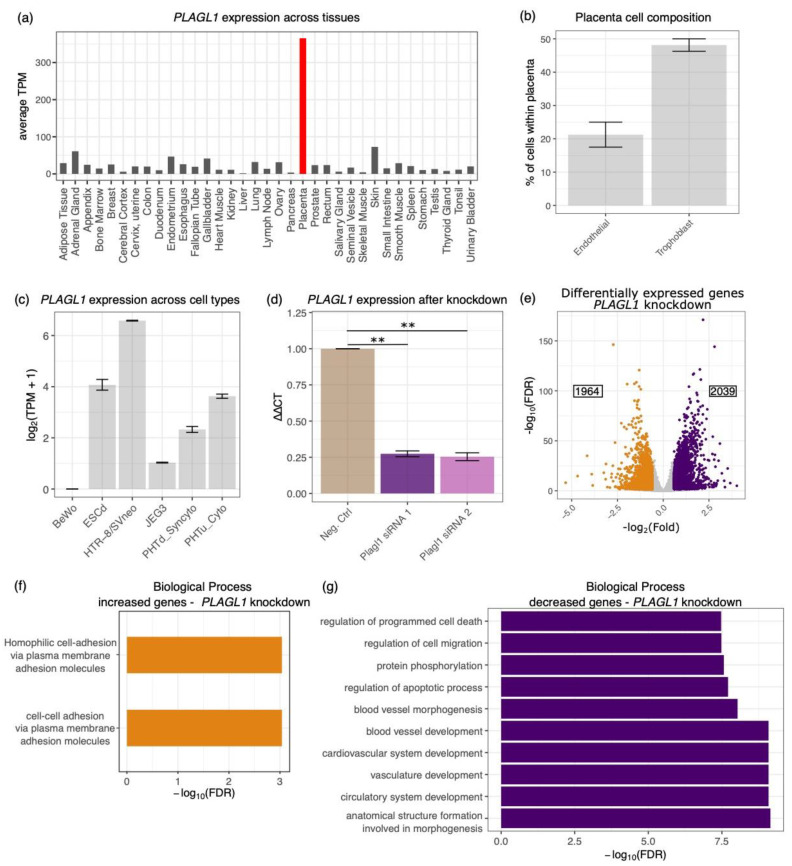
PLAGL1 regulates genes involved in blood vessel development. (**a**) Bar graph of PLAGL1 expression in human tissues, generated using TissueEnrich. Expression reported using transcripts per million (TPM). (**b**) Percentage of trophoblast and endothelial cells in human placental samples. Data is from the Human Protein Atlas. (**c**) Bar graph of PLAGL1 expression in multiple human cell lines. (**d**) *PLAGL1* expression is significantly reduced by two siRNAs (*p*-value ≤ 0.01 (**)) compared to a negative control. Values are normalized to the negative control siRNA. (**e**) 4003 genes are differentially expressed after *PLAGL1* knockdown in HTR-8/SVneo cells. Genes which increase (1964) in expression are indicated as orange dots on the volcano plot and those that decrease (2039) are purple. (**f**) Cell-adhesion terms are associated with genes that are upregulated when *PLAGL1* is knocked down. (**g**) Vasculature development terms are associated with genes that are downregulated when *PLAGL1* is knocked down.

We next investigated a potential role for PLAGL1 in human placenta. Using data from the human protein atlas [48] and the TissueEnrich tool [49], we found that *PLAGL1* has placenta-enriched gene expression (Figure 5a). The human protein atlas further showed that the placenta sections used for analysis were comprised primarily of trophoblast cells (Figure 5b). Therefore, we sought to investigate the role of *PLAGL1* in human trophoblast cells. First, we evaluated RNA-seq data from several human cell lines [50] for *PLAGL1* expression, including: choriocarcinomas representing villous trophoblast (BeWo [51] and JEG3 [52]); syncytiotrophoblast (PHTd_Syncytio [53]) differentiated from term placenta cytotrophoblast (PHTu_Cyto [53]); BAP treated human embryonic stem cells (ESCd [53]); and a cell line derived from chorionic villi explants of first trimester placenta (HTR-8/SVneo [54]). We proceeded with our experiments in HTR-8/SVneo cells, since they had the highest expression of *PLAGL1* (Figure 5c). When grown on Matrigel, HTR-8/SVneo cells express genes associated with blood vessel development, can form endothelial tube-like structures, and are commonly used to model endovascular differentiation of extravillous trophoblast [55,56,57,58,59].

To understand the global impacts PLAGL1 has on gene expression, we performed an siRNA knockdown of *PLAGL1* followed by RNA-seq in the HTR-8/SVneo cells. We first tested two siRNAs, and found that both siRNAs knocked down *PLAGL1* by 73–75% on average (Figure 5d). Since both siRNAs showed similar knockdown efficiencies, we proceeded with RNA-seq using siRNA 1. We identified 4003 genes that were differentially expressed (fold ≥ 1.5, adjusted *p*-value ≤ 0.05) between the *PLAGL1* and negative control knockdown samples using DESeq2 [60] (Figure 5e; Supplemental Table S4). The 1964 genes that were increased upon *PLAGL1* knockdown included several protocadherins such as *PCDH1*, *PCDH10*, and *PCDH7*. The only terms enriched in this group were related to cell-cell adhesion (Figure 5f). On the other hand, the 2039 genes that decreased as a result of *PLAGL1* knockdown were enriched for terms related to blood vessel development, cell migration, and both type 1 (insulin dependent) and type 2 (non-insulin dependent) diabetes (Figure 5g; Supplemental Figure S3).

**Figure 6:**
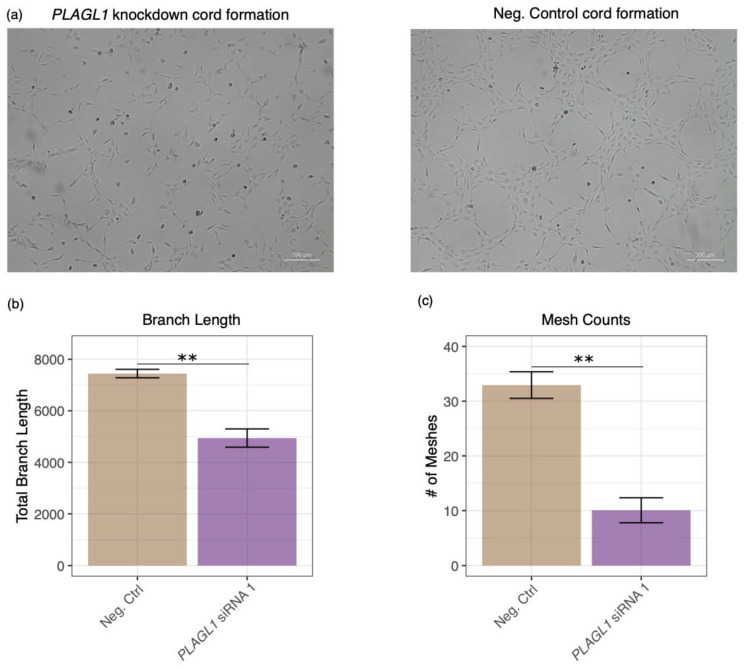
*PLAGL1* knockdown decreases cord formation ability in HTR-8/SVneo cells. (**a**) Representative images showing cord formation is reduced after PLAGL1 is knocked down (left) compared to a control (right). (**b**) Branch length was significantly reduced after PLAGL1 knockdown (*p*-value ≤ 0.01 (**)). (**c**) Enclosed regions, or meshes, were significantly reduced after PLAGL1 knockdown (*p*-value ≤ 0.01 (**)).

To determine if *PLAGL1* could play a role in the ability of HTR-8/SVneo cells to mimic endothelial cell tube-like structure formation, we performed a tube formation assay. Ten hours after plating the cells transfected with the *PLAGL1* siRNA, we see a significant decrease in cord formation, as determined by the total branching length (*p*-value = 0.0093) and number of enclosed regions (meshes; *p*-value = 0.0024) compared to cells transfected with a negative control (Figure 6).

### 2.6. Relationship between Mouse and Human Plagl1 Results

The gene networks regulated by PLAGL1 in mouse placenta, where it is expressed in endothelial cells, and in HTR-8/SVneo human trophoblasts are at least partially conserved. For example, the GO terms enriched for genes downregulated upon *PLAGL1* knockdown in HTR-8/SVneo cells were similar to the terms associated with the genes we initially predicted to contain PLAGL1-binding motifs in their enhancer regions in the mouse genome (Figure 4a and Figure 5g). Therefore, we tested whether the specific genes associated with terms enriched for predicted PLAGL1 enhancers decreased in expression after *PLAGL1* was knocked down in HTR-8/SVneo cells. To do this, we focused on the terms that were associated with *PLAGL1* enhancers—‘abnormal placental labyrinth vasculature morphology’, ‘regulation of cellular response to insulin stimulus’, and ‘placental development’. Of the eight target genes associated with ‘abnormal placental labyrinth vasculature morphology’, four were downregulated upon *PLAGL1* knockdown in HTR-8/SVneo cells (*p*-value of overlap = 0.00188). Of the five predicted PLAGL1 target genes associated with ‘regulation of cellular response to insulin stimulus’, three were downregulated upon *PLAGL1* knockdown in HTR-8/SVneo cells (*p*-value of overlap = 0.00161). Of the 11 predicted PLAGL1 target genes associated with the more general term, ‘placental development’, five were downregulated upon *PLAGL1* knockdown (*p*-value of overlap = 0.00159) (Supplemental Figure S4a).

We further determined if the enhancers identified in mouse could drive gene activity in the HTR-8/SVneo cells. We performed a dual-glow luciferase assay using five enhancers predicted to be bound by PLAGL1 and target genes with varying functions, including blood vessel development (COL1A1 [18]), migration (DLC1 [61]), signal transduction (ARHGEF3 [62]), insulin regulation (IRS1 [63]) and fetal growth (IGF2BP1 [64]). We found that all of these regions were indeed acting as enhancers in the HTR-8/SVneo cells (relative luciferase activity ≥ 2) (Supplemental Figure S4b). To confirm PLAGL1 regulates the activity of these enhancers, we tested the activity with and without an siRNA-mediated knockdown. After *PLAGL1* knockdown, the enhancer activity significantly decreased in three out of five of these enhancers (Supplemental Figure S4c).

## 3. Discussions

By combining RNA-seq and ChIP-seq data from the mouse placenta, we identified *Plagl1* as an upregulated transcription factor that has its motif enriched within e9.5-specific enhancers. In mouse, gene ontology analysis showed that PLAGL1 was predicted to associate with genes involved in labyrinth layer development and fetal growth. *Plagl1* was expressed throughout the allantois and within vascular endothelial cells of the labyrinth layer. We also found that *Plagl1* has sex-specific gene expression differences between normal placentas and those from the GDM mouse model. Next, we performed an siRNA-mediated knockdown of *PLAGL1* in HTR-8/SVneo cells to determine if the gene could be important in human placental trophoblast cells. Ontology analysis of genes which decreased in expression upon *PLAGL1* knockdown showed enrichment of terms related to blood vessel remodeling. *PLAGL1* was further implicated in this role by a tube formation assay, where we observed a decrease in cord formation in *PLAGL1* knockdown cells.

In mouse, the importance of PLAGL1 in fetal growth has been established, as pups from global PLAGL1 knockout mice are smaller in size compared to wild type pups. The authors found a slight decrease in placental weight by gestational day 16.5. However, they did not report a difference in the histology of the placenta or its ability to transport glucose [65]. Our analysis identified a PLAGL1 motif in e9.5 placenta enhancers predicted to target *Ppar*γ and *Soc3.* Both genes are associated with ‘regulation of cellular response to insulin stimulus’ and ‘abnormal placental labyrinth vasculature morphology’ (Supplemental Figure S4a). These genes were also found to significantly decrease in HTR-8/SVneo cells after *PLAGL1* knockdown and are a part of terms like ‘blood vessel development’. Interestingly, PLAGL1 has been associated with the upregulation of each of these genes in beta cell proliferation, and insulin secretion or signaling, and both genes have been suggested as targets of study to better understand the role of PLAGL1 in transient neonatal diabetes [66]. However, such a relationship has not been established in placental vasculature although PPARγ null placentas, and SOCS3 null placentas both show defects in labyrinth formation and maternal blood sinuses [67,68]. Although we predict other targets of PLAGL1 that may be involved in blood vessel formation, future work, including PLAGL1 ChIP-seq, is necessary to confirm predicted, and identify novel, PLAGL1 binding sites. Given the association between PLAGL1 and angiogenic gene expression in the mouse placenta prior to major endovascular invasion, a role in blood vessel formation within the labyrinth layer is also possible. To test this, placenta-specific knockout of PLAGL1 would need to be generated, since *Plagl1* is highly expressed in other mouse embryonic tissues, including the liver and limb [69].

Our findings also revealed that, amongst male placentas, *Plagl1* is more highly expressed in a mouse model of GDM compared to controls. This adds to the evidence that *Plagl1* methylation and expression are sensitive to the maternal environment. *Plagl1* is hypomethylated and its expression is upregulated in mouse offspring generated by assisted reproductive technologies, and its methylation is associated with maternal folate concentrations, a Mediterranean diet, alcohol, and vitamin B2 consumption in women [70,71,72,73]. These *Plagl1* alterations are associated with birthweight and childhood obesity [70,74], and similarly, offspring of the mouse model of GDM utilized here have greater adiposity, and are more sensitive to metabolic disruption of their reproductive systems [75,76,77]. However, it is not clear whether placental *Plagl1* plays a functional role in these offspring outcomes, or is simply a marker for them; it is a putative metastable epiallele, meaning that environmental alterations in placental gene regulation may be maintained in offspring tissues and affect their adult functions [74]. *Plagl1* knockdown in the human trophoblast cells shows altered expression of *Oas1* and *Polr2g,* which are misexpressed in placentas from women with GDM, in the direction that would be predicted by PLAGL1 overexpression (downregulated and upregulated, respectively), suggesting a functional role for PLAGL1 in GDM-induced placental dysfunction [78]. Moreover, the findings here suggest that *Plagl1* misexpression could contribute to offspring outcomes in GDM by directly regulating placental function. For example, placental capillary density is increased, and branching decreased, in placentas from GDM pregnancies [44], consistent with the potential role of *Plagl1* in angiogenesis that we have uncovered. However, further experiments are needed to directly test whether placental angiogenesis is altered in this murine model of GDM, and whether *Plagl1* is responsible.

Notably, the effect of GDM on placental *Plagl1* expression in this model is sex-specific occurring in males only. In contrast, low birthweight is only associated with *Plagl1* methylation changes in female placentas [74]. Other genes have also been shown to be differentially expressed between male and female placentas associated with GDM, including lipolipase, which is involved in fatty acid transport and uptake [79]. Interestingly, GDM, like PLAGL1, is known to have sex-specific attributes. GDM is more common in pregnancies with a male fetus [80] and is also a risk factor for later type 2 diabetes of the fetus, with males developing it more than females [81]. GDM has also been shown to have different effects on thyroid hormone receptors within the placenta for male and female placentas [82], and affect glucose utilization differently between the sexes [83]. The mouse model of GDM used here affects the metabolism of both male and female offspring, but more dramatically impacts adipose tissue gene expression and substrate utilization in males [76].

The HTR-8/SVneo cells used for knockdown and subsequent experiments were of female origin [84]. Due to the sex-specific responses and expression of *Plagl1*, it would be advantageous to check the results in a male cell line to determine if PLAGL1 regulates the same genes in both genders. Much work remains in understanding the impact of sex on PLAGL1 functions and regulation.

Not only can PLAGL1 have sex-specific roles, but PLAGL1 is known to have different, or even opposite, functions depending on the type of cells being studied and on the co-factors that are expressed with it. For example, PLAGL1 induces activation of the PAC1 receptor promoter, unless co-transfected with ERA, which then leads it to repress promoter activity [37]. PLAGL1 expression has also been found to be increased in some tumor tissues [85,86], and decreased in others [87,88]. Since PLAGL1 can have different effects in different environments, it would be interesting to compare the effects of *PLAGL1* knockdown in specific subsets of trophoblast cells. Recently, human placental cells were cultured and differentiated into extravillous and syncytiotrophoblast cells [89], which have varying degrees of *PLAGL1* expression. Knockdown and functional assays on multiple trophoblast subtypes will give us a greater insight into the diverse functions and cell-specific roles of PLAGL1. Other cell lines that could also be more appropriate for studying aspects of blood vessel formation, and that could help better understand the results we observe in mouse, include human placental vascular endothelial cells (HPVEC) or human umbilical vein endothelial cells (HUVEC).

In HTR-8/SVneo cells, we found that genes downregulated upon *PLAGL1* knockdown were strongly enriched for blood vessel development terms. Interestingly, both HTR-8/SVneo cells and primary first trimester trophoblast, but not third trimester trophoblast, are capable of cord formation [90], indicating that this property is characteristic of trophoblast cells capable of endovascular invasion. We found that by knocking down *PLAGL1,* cord formation of HTR-8/SVneo cells was hindered. However, to determine if PLAGL1 has a role in tube formation, other methods such as co-culture assays would need to be assessed, since several non-endothelial cells can also form tubes in the presence of Matrigel [91]. Genes upregulated upon *PLAGL1* knockdown were enriched for cell adhesion terms. Previous research has found that PLAGL1 targets genes involved in cellular adhesion and extracellular matrix composition [92,93]. We also observed that several immune genes were upregulated including interferons (*IRF6*, *IRF7*) [94,95], *TGFB3* [96], and *HLA-DQB1* [97]. However, the role of PLAGL1 in immunity is unknown. Further analysis could reveal a role of PLAGL1 in regulating an immune response in the placenta.

Although we performed experiments and bioinformatics analysis in both mouse placenta and a human trophoblast cell line that represent different aspects of trophoblast-endothelial interactions in placental development, we do observe similarity of PLAGL1 targets and predicted functions, as noted above. In mouse, however, we observed *Plagl1* expression in endothelial cells, whereas in human we investigated trophoblast cells. It is possible PLAGL1 has a role in both cell types in both species, and we could not capture this with the specific timepoint we investigated in mouse, and the specific cell line we used in human. We note, however that there are also similarities in the genes identified in our study and PLAGL1 target genes identified in other studies. For example, a previous study analyzed 15 imprinted genes associated with *Plagl1* and checked if they displayed significantly different expression in the embryonic liver (e18.5) of WT mice compared to the liver in *Plagl1* knockout mice [65]. Of these 15 imprinted genes, we found that *CDKN1C*, *DCN*, *GATM*, *GRB10*, *MEG3*, and *MEST* were upregulated in the HTR-8/SVneo *PLAGL1* knockdown cells, while others, including *SLC38A4*, *IGF2*, and *IGF2R* have downregulated family members in the HTR-8/SVneo cells. We found other known imprinted targets of PLAGL1 to be disrupted in the HTR-8/SVneo *PLAGL1* knockdown cells, such as *RASGRF1* [98]. *TP73* and *WT1*, which were also misregulated upon *PLAGL1* knockdown, were previously found to be highly methylated in prostate cancer, along with *PLAGL1* [99].

Although many roles of PLAGL1 have been well studied in different tissues and cells, including its ability to regulate migration [100] in neurons and proliferation [38] in several tissues, a role in the placenta has not been thoroughly described. We provide evidence that PLAGL1 alters angiogenic gene expression and function. Proper remodeling of maternal blood vessels and formation of fetal blood vessels are critical for efficient transport of nutrients and oxygen between the maternal and fetal blood supply. Errors in this process could lead to several complications. Therefore, PLAGL1 may be an interesting target of analysis when understanding the pathogenesis of pregnancy diseases.

## 4. Materials and Methods

### 4.1. Filtering of e7.5 and e9.5 Placenta RNA-seq Data

We obtained previously analyzed and published e7.5 and e9.5 placenta RNA-seq data from the Gene Expression Omnibus (GSE65808) [13]. TFs were identified from the Animal Transcription Factor Database(v2.0) [101]. TFs were considered highly expressed and retained for analysis if they had an FPKM ≥ 10 at e9.5 and were significantly upregulated compared to e7.5 (fold ≥ 2 and *q*-value ≤ 0.05).

### 4.2. Binding Site Predictions

To identify the potential binding sites of the highly expressed TFs, we used a curated library of position weight matrices as previously described [28], only retaining those with high information content (≥10). Using the PRISM phylofootprinting method [28], we predicted binding sites for the highly expressed TFs in previously defined e9.5-specific enhancers, downloaded from the Gene Expression Omnibus (GSE65807) [13]. We filtered the predictions using the parameters as described in [102], only keeping significant (*p*-value ≤ 0.05) predictions with a match threshold of at least 0.8 that are conserved in the human genome (hg19).

Motifs with a similarity threshold of 0.8 were grouped, and only the motif with the highest number of occurrences was kept for further analysis. We determined the number of e7.5 and e9.5 enhancers with each motif and then calculated the fold by determining the proportion of enhancers containing a particular motif at e9.5 divided by the proportion at e7.5. Significance was determined using the hypergeometric test with Bonferroni correction for multiple comparisons.

### 4.3. Plagl1 Timepoint Expression qPCR

Animal experiments for this qPCR and for RNA-Scope were approved by the Iowa State University Institutional Animal Care and Use Committee (Protocol IACUC-18–350) and conformed to the NIH Guidelines for the Care and Use of Laboratory Animals. Placenta tissue was collected from e7.5 and e9.5 timed-pregnant mice, as described previously [13], for a total of three biological replicates per timepoint. For e7.5, 12 placentas were collected per biological replicate and for e9.5, one placenta was collected per biological replicate in 500 ul PBS supplemented with 1X protease inhibitor. RNA was isolated using the Purelink RNA Micro Scale kit (Thermofisher Scientific 12183016) following the recommended protocol for purifying RNA from animal tissue. Isolated RNA concentrations were measured using the Nanodrop lite (Thermofisher Scientific ND-LITE, Waltham, MA, USA) and 200–400 ng of RNA was converted into cDNA for each biological replicate with the Applied Biosystems cDNA Reverse Transcription kit (Thermofisher Scientific 4368814). *Plagl1* expression was quantified by RT-qPCR on the CFX connect real time PCR system (BioRad 1855201) with 6ng of cDNA per biological replicate. *Polr2a* was used for normalization of *Plagl1* expression (ΔCT). Primer sequences and efficiencies can be found in Supplemental Table S5.

### 4.4. RNA-Scope

RNAscope was performed by the Comparative Pathology Core at Iowa State University College of Veterinary Medicine using the RNAscope 2.5 High Definition (HD)-Red Assay (Advanced Cell Diagnostics Cat #322350) according to manufacturer’s instructions. Briefly, for each biological replicate, e9.5 implantation sites were dissected from timed-pregnant CD-1 mice obtained from Charles Rivers Labs. Each implantation site was embedded in paraffin and was cut at 5 μm thickness, mounted, deparaffinized and treated with hydrogen peroxide prior to target retrieval, and then treated with protease plus. Mm-Plagl1 (cat # 462941) and control probes were applied to slides for hybridization followed by six rounds of amplification. The signal was detected with Fast RED and slides were counterstained with hematoxylin. *Plagl1* signal appears as red on the slide. Control slides included positive control probe Pentidylpropyl isomerase B (Mm-PPIB cat # 313911) used to determine RNA viability in the samples, and negative control probe DapB (cat # 310043) to detect nonspecific staining.

### 4.5. Immunohistochemistry Staining

Paraffin embedded tissue sections, collected as described in the RNA-scope methods section, were incubated at 60 °C for 20 min. Immediately after incubation, the sections were deparaffinized with three washes in xylene for 5 min each, followed by three washes in 100% ethanol for 3 min each, one wash in 95% ethanol for 1 min and rinsing the slide under running deionized water (DI) for 3 min. Antigen retrieval was achieved by placing the slides in sodium citrate buffer (pH 6) for 40 min at 100 °C in a water bath. The slides, along with sodium citrate buffer were allowed to cool down to room temperature (RT) for 30 min and washed two times in PBST buffer (0.1% tween20 in PBS pH 7.4) for 5 min each wash. The sections were blocked with hydrogen peroxide (Fisher scientific NC0185217) for 10 min and washed two times with PBST buffer, 5 min each. The sections were incubated at RT for 1 h in blocking buffer (1% DMSO and 1% BSA in PBS buffer). After draining the blocking buffer, primary staining was performed using the CD34 antibody (Abcam ab81289, Cambridge, UK) at a 1:400 dilution in blocking buffer, and slides with primary antibody were incubated overnight at 4 °C in a humidified chamber. Following primary antibody staining, the sections were washed in six changes of PBST for 5 min each and incubated for 1 h at RT in secondary Goat anti-rabbit HRP antibody (Abcam ab6112) at a 1:750 dilution in blocking buffer. This was followed with three washes in PBST, for 5 min each. The staining was visualized using DAB for 10 min (Fisher scientific NC9276270, Hampton, NH, USA) following the vendors recommended protocol and washing away excess DAB under running DI water for 5 min. The sections were counter stained with Mayer’s Hematoxylin (Fisher scientific 5031794) for 7 min at RT and incubated in bluing agent (Fisher scientific 22050114) for 2 min. The sections were washed in DI water and patted dry before mounting.

### 4.6. GDM Placenta qPCR

All animal procedures were approved by the Baylor College of Medicine institutional animal care and use committee and performed in accordance with NIH Guide for the Care and Use of Laboratory Animals. Seven week old C57BL/6J female mice were placed on either a 10% kcal/fat, 0% kcal/sucrose control diet (Ctrl) or a 45% kcal/fat, 17% kcal/sucrose diet (GDM) one week prior to and throughout pregnancy to induce GDM like symptoms as previously described [45]. All females were placed with a proven breeder male for one night and then examined for copulatory plugs in the morning. Plug positive females were considered pregnant and morning of plug positive was designated as day 0.5 of pregnancy. At embryonic day 17.5, dams were euthanized. For GDM mice, 10 placentas were dissected from 5 dams. For control mice, 11 placentas were dissected from six dams. Placenta were individually flash frozen. Flash frozen placenta were thawed overnight in RNA*later*-ICE and RNA extracted using PureLink RNA Mini Kit. Quality of RNA was checked on a 1% Agarose gel. RNA was converted to cDNA using the High-Capacity cDNA Reverse Transcription kit (ThermoFisher Scientific 4368814). Primers used for *Plagl1* and *Polr2a* (mouse) can be found in Supplemental Table S5.

Sex of the placenta was determined as previously described [103] using PCR to amplify either *Rbm31x* and *Rbm31y*. Samples were then run on a 1% agarose gel to determine sex (Supplemental Table S3).

### 4.7. TissueEnrich Analysis

*PLAGL1* expression data for human tissues was obtained from the ‘Tissue-specific Genes’ option of the TissueEnrich [49] webtool. Expression values were based on data from the Human Protein Atlas.

### 4.8. Human Protein Atlas Placenta Analysis

To obtain the percentage of trophoblast and endothelial cells in human placenta samples used for experiments, we used the eight available samples (sample IDS: 200, 202, 203, 204, 375, 385, 398, 413) from the Human Protein Atlas [48]. Cell percentages were averaged across samples.

### 4.9. siRNA knockdown and qPCR

HTR-8/SVneo (ATCC^®^ CRL3271™) cells were cultured in RPMI-1640 (ATCC 30-2001) with 5% FBS. Cells were seeded at 15 × 10^4^ cells/well in a 6-well plate and grown for 48 h before transfecting. Transfections were carried out with the Lipofectamine RNAiMAX Transfection Reagent (Fisher Scientific 13778150) and with *PLAGL1* siRNAs (ThermoFisher Scientific 4392420-s10602, s10603) or a negative control (ThermoFisher Scientific 4390843). We performed a media change 24 h after transfection. RNA was extracted 48 h after transfection using the Qiagen Mini RNA kit and quality was determined using the Bioanalyzer Total RNA nano analysis kit (Agilent, Santa Clara, CA, USA) and all RNA Integrity Numbers were greater than 8. Concentrations were determined using the Nanodrop and 1000 ng of RNA from eight biological replicates was collected for RNA-seq. 400 ng of RNA was then converted to cDNA using the High-Capacity cDNA Reverse Transcription kit (ThermoFisher Scientific 4368814). *PLAGL1* knockdown was quantified by Real-Time qPCR. *GAPDH* was used for normalization and percent knockdown of *PLAGL1* was calculated using the ∆∆CT method. Primer sequences and efficiency values can be found in Supplemental Table S5.

### 4.10. HTR-8/SVneo RNA-seq

Libraries were prepared for all eight replicates by the Iowa State DNA facility using the NEBNext Ultra II Directional library prep kit, unique dual index plate, and poly(A) mRNA magnetic isolation module, following manufacturer’s protocol. Samples were run on the HiSeq 3000, across three lanes using single-end 50 bp reads. Reads were combined across lanes, aligned to the hg19 genome using HISAT2 [104] (v2.1.0; default parameters) (Supplemental Table S6), and transcript abundance was calculated using htseq-count from the HTseq [105] package. Significantly differentially expressed genes were identified using DESeq2 [60] (1.28.1, default parameters) and defined as genes with a fold of at least 1.5 and FDR ≤ 0.05. All raw and processed RNA-seq data have been made available in the GEO repository, under the data accession GSE154577.

### 4.11. Tube Formation Assay

Tube formation assays were performed as previously described with minor modifications [106]. 150 μL of Matrigel (Fisher Scientific CB40234A) was used to coat wells of a 24-well plate and solidified at 37 °C for 1–2 h. Cells were seeded at a density of 75,000 cells/well in RPMI-1640 media, 48 h after treatment with the *PLAGL1* siRNA or a scrambled control. Images were taken after 10 h of incubation on the Matrigel using light microscopy from three random fields of each well. Cord formation ability was calculated by counting the number of meshes and the total branch length using the Angiogenesis Analyzer plugin from ImageJ.

### 4.12. Cloning

Primers to amplify putative PLAGL1 enhancer regions were designed using the mm9 genome and cloned into the PGL4.23 vector using the ligation independent cloning method as previously described [107] with a modification. Target enhancer regions were amplified with NEB Q5 DNA polymerase (NEB M0491S) using primers listed in Supplemental Table S7. For each enhancer target, three colonies were selected to identify positive clones by colony PCR and sequenced with Applied Biosystems 3730xl DNA Analyzer (DNA Facility, Iowa State University).

### 4.13. Enhancer Testing with a Dual Glow Luciferase Assay

HTR-8/SVneo cells were seeded at 50,000 cells/well and grown for 24 h on a 24-well plate before being transfected with *PLAGL1* or the negative control siRNA. 24 h after this, cells were transfected again using Jetprime reagent (VWR 89129-924) with a control plasmid or an enhancer cloned into the PGL4.23 vector. The following day the plates were read following the manufacturer’s protocol for the Dual-glow Luciferase Assay kit (Fisher Scientific PRE2920). To determine the significance of the relative luciferase assay change, firefly luciferase values were normalized to renilla luciferase values and then the knockdown was compared to negative control values for the same replicate using a *t*-test. Experiments were run with four biological replicates for siRNA 1.

### 4.14. Ontology Analysis

Gene ontology enrichment analysis for enhancers was carried out using GREAT (v3.0.0) [25] with the GO biological process, mouse phenotype, and disease ontologies. Default parameters were used for region-gene associations, unless otherwise specified, and all analyses show top terms, ranked by FDR. Ontology enrichment for sets of genes was carried out using WebGestalt using over-representation analysis and the biological process database, unless otherwise specified. The reference set for comparison was the protein-coding genome and other settings were left as default. All analyses show top terms, ranked by FDR.

### 4.15. Statistical Analysis

Experiments were repeated in triplicate and results are displayed as the mean ± SE unless otherwise indicated. *p*-values were calculated using student’s t-test unless otherwise specified.

## Supplementary Materials

The following are available online at https://www.mdpi.com/1422-0067/21/21/8317/s1, Figure S1: Transcription factor analysis, Table S1: E9.5-specific enhancer gene ontology terms, Figure S2: RNAscope showing *Plagl1* expression in the allantois and labyrinth, Table S2: E9.5 highly expressed, upregulated transcription factors, Figure S3: Disease ontology for downregulated genes, Table S3: Samples used for *Plagl1* expression in GDM placentas, Figure S4: PLAGL1 target gene and enhancer activity, Table S4: DESeq2 output, Table S5: List of primers used for RT-qPCR, Table S6: HTR-8/SVneo HISAT2 results, Table S7: Target enhancer primers.

## Abbreviations

e	Embryonic day
TF	Transcription factor
PLAGL1	pleiomorphic adenoma gene-like 1
GDM	gestational diabetes mellitus
FPKM	fragments per kilobase of transcript per million of mapped reads
TPM	transcript per million
GREAT	Genomic Regions Enrichment of Annotations Tool
Ctrl	Control diet
