# Supplementary material for: Transcription Factor PLAGL1 Is Associated with Angiogenic Gene Expression in the Placenta
Source: Int J Mol Sci. 2020 Nov 6;21(21):8317. doi: 10.3390/ijms21218317 (PMC7664191; doi:10.3390/ijms21218317)
Supplement: Supplementary file 1 [file ijms-21-08317-s001.zip › supplementaryMaterial/Starks_SupplementalFigures_V5.docx]

Supplemental Figure 1

(b)

(a)

**Supplemental Figure 1. (a)** Transcription factor analysis. Diagram depicting the number of genes remaining after each filtering step, resulting in 37 TFs considered for motif enrichment analysis in e9.5-specific enhancers. **(b)** *Plagl1* expression at e7.5 and e9.5 in the placenta. Bar chart shows significantly higher expression at e9.5 (p-value ≤ 0.05(*)).

Supplemental Figure 2

**Supplemental Figure 2.** RNAscope showing *Plagl1* expression in the allantois and labyrinth. **(a)** Second biological replicate of RNAscope showing *Plagl1* RNA expression in developing labyrinth and the allantois (dark red) of the e9.5 placenta. Box shows zoomed in region (right) and arrowheads indicate *Plagl1* staining of endothelial cells. **(b)** RNAscope positive control staining of PPIB. **(c)** RNAscope negative control staining DapB. **(d)** Second replicate of immunohistochemistry showing CD34 staining the vascular endothelial cells. Box shows zoomed in region (right) and arrowheads indicate CD34 staining.

Supplemental Figure 3

Disease Ontology

decreased genes – *PLAGL1* knockdown

**Supplemental Figure 3.** Disease ontology for downregulated genes**.** Disease Ontology terms associated with genes that decrease in expression when *PLAGL1* is knocked down.

Supplemental Figure 4

(a)

(c)

(b)

*

*

Enhancer activity after

*PLAGL1* knockdown

Enhancer activity

*

**Supplemental Figure 4.** PLAGL1 target gene and enhancer activity. **(a)** Gene information for three enriched terms associated with e9.5-specific enhancers containing a PLAGL1 binding motif. All genes listed in the column are a part of the term below it. Genes that are associated with e9.5-specific enhancers containing a PLAGL1 binding site that are also downregulated in HTR-8/SVneo cells upon *PLAGL1* knockdown are in white text (dark purple bar). Genes that are associated with e9.5-specific enhancers containing a PLAGL1 binding site that are not downregulated in HTR-8/SVneo cells upon *PLAGL1* knockdown are in black text (light purple bar). **(b)** Bar graph of luciferase activity for five enhancers containing a putative PLAGL1 motif. All five represent active enhancers (relative luciferase activity ≥ 2 (black line)). **(c)** Bar graph of luciferase activity for enhancers from (b) with and without a *PLAGL1* knockdown. Several enhancers show a significant decrease in activity (p-value ≤ 0.05(*); 1-tailed t-test).
